# Mapping quality of life after balloon dilatation in subglottic stenosis using Dyspnea index and Short Form Health Survey-36

**DOI:** 10.1007/s00405-024-08667-0

**Published:** 2024-05-06

**Authors:** Anders Erlandsson, Anders Lundquist, Katarina Olofsson

**Affiliations:** 1 Department of Clinical Sciences, Otorhinolaryngology, Umeå University, Umeå, Sweden; 2 Umeå School of Business, Economics, and Statistics, Umeå University, Umeå, Sweden; 3 Department of Otorhinolaryngology, University Hospital of Umeå, 901 89 Umeå, Sweden

**Keywords:** Subglottic stenosis, Balloon dilatation, Dyspnea index, Short form healthy survey

## Abstract

**Purpose:**

An accurate diagnosis and proper treatment plan are required to restore an adequate patent airway in fibrotic subglottic stenosis (SGS). Currently, the definitive treatment entails single-stage balloon dilatation with steroid injections. The primary aim was to evaluate successful airway restoration and general quality of life in cases with SGS in northern Sweden using robust patient reported outcomes.

**Methods:**

All participants with need of surgical treatment due to SGS that had been referred to the department of otorhinolaryngology, University Hospital of Umeå from September 2020 to August 2023 was included. Exclusion criteria included malignant, extrathoracic or cartilaginous cause, age < 18 years, or incompetent to sign consent documents. We assessed the patient-reported outcome measures pre- as well as 3 months postoperatively.

**Results:**

Of the 40 cases fulfilling the eligibility criteria’s, 33 cases completed the Dyspnea index (DI) and the short form health survey (SF-36) pre- as well as 3 months post-operatively. Receiver operating characteristics showed significant improvement in DI as well as in SF 36 scores post-operatively.

**Conclusions:**

Evaluation of balloon dilatation in SGS in this cohort follow-up analysis shows clear improvement in patient quality of life using robust PROM 3 months postoperatively, ensuring the use of a safe and well-tolerated procedure.

## Introduction

The inability to breathe normally is a severe disabling symptom with high impact on quality of life. Subglottic stenosis (SGS) is as a fibrotic devasting disease of unclear cause, located just below the vocal folds. SGS progressively obstructs the upper airway, there is no cure and thereby a recurring need of symptom-relieving surgical treatment. SGS is heterogeneous in its clinical symptomatology, and as such mis-, and delayed diagnoses are frequent, usually due to confusion with other respiratory diseases, even after comprehensive workup [1, 2]. The mean time to diagnosis has been reported to be more than 2 years from the onset of symptoms [1–3]. Including all phenotypes the incidence is 0.49/100 000 annually. Although relatively uncommon, SGS is highly relevant from an economic perspective with annual healthcare costs comparable to COPD and diabetes mellitus [4, 5].

The SGS pathogenesis is not completely clear but can include mucosal damage caused by prolonged or traumatic intubation or autoimmune disorders such as granulomatosis with polyangiitis. In some cases, the cause of SGS can remain unidentified despite a thorough evaluation, termed as idiopathic [6]. The idiopathic type of SGS is rare, with an incidence of 1/200,000, affecting predominantly otherwise healthy perimenopausal females of Caucasian origin [3].

Given that recurrence of SGS is regarded as the natural course of the condition, the main treatment goal is symptomatic relief, to restore airway patency. SGS can be treated surgically through endoscopic procedures using balloon dilatation, CO_2_ laser, or a combination of these. Open surgical approaches have been utilized in more complicated stenosis mainly of cartilaginous origin [7]. Medical treatment is an option both by itself or in combination with endoscopic procedures [8].

Because of the multiple possible causative factors and the lack of a golden standard treatment protocol, the evaluation of quality-of-life aspects is important to assess treatment efficacy for SGS patients. Use of patient reported outcome measures (PROMS) such as the Dyspnea index (DI) and short form health survey (SF-36) has been proven reliable in previous studies and has also been correlated to more objective airway measuring, such as peak expiratory flow (PEF%) [9–11]. DI is a PROM form specifically targeting upper airway diseases that recently has been validated in Swedish and used in evaluation of SGS [10, 12]. SF-36 is a well-accepted and widely used PROM form which takes into account both the patient physical as well as psychological well-being [11]. The reason for using PROMS as measures of effect from surgery is that it is trying to capture what is experienced or perceived by patients, thus avoiding interpretation bias by caregivers. This study aims to evaluate the treatment outcomes of the currently used endoscopic balloon dilatation technique in SGS utilising two validated PROM’s pre- and post-operatively. Being a safe and worldwide used procedure, we hypothesise significant improvement in quality of life in the treated cases in Northern Sweden.

## Materials and methods

### Ethical approval

All procedures were performed in accordance with the 1964 Declaration of Helsinki and its later amendments. The study was approved by the Swedish Ethical Review authority, approval number nr; 2020-00253 (2020-04-14). We confirm that all methods were performed in accordance with the relevant guidelines and regulations according to the Strengthening the Reporting of Observational studies in Epidemiology (STROBE) checklist.

### Study design

Prospective descriptive, cross-sectional.

### Data collection

#### Participant recruitment

**Fig. 1:**
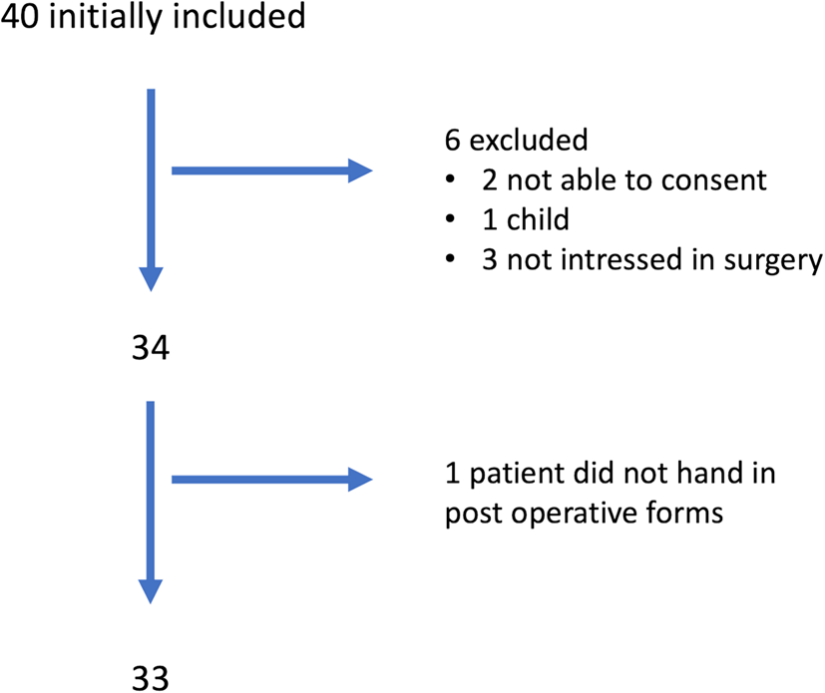
Flowchart showing excluded cases

Forty (40) adult patients with newly or recurrent SGS who were scheduled to undergo endoscopic treatment at the department of otorhinolaryngology at University Hospital of Umeå between September 2020 and August 2023 fulfilled the eligibility criterions. Thirty-three study participants completed the Swedish version of the DI and SF-36 forms. The potential participants were identified by symptoms of respiratory distress, flexible endoscopy (awake) and CT (computer tomography)-scan. For this study, subglottic stenosis was defined as a fibrous stenosis at the level of the cricoid cartilage. Exclusion criteria were a malignant, extra thoracal, or cartilaginous cause, < 18 years of age, multilevel extension, incomplete clinical documentation, missed clinical follow-up or not competent to provide consent. Table 1 shows the demographic data of the study population at baseline including sex, age, smoking, and body mass index. Other respiratory diseases were not classified or reported here, where they did not affect the anaesthetic procedure nor the care of the patients. Figure 1 shows the flowchart for inclusion and exclusion.

**Table 1:** Demographic data of the study population at baseline. *BMI* body mass index scores between 25 and 30 is defined as overweight, > 30 as obesity

Demographics	*n* = 33 In brackets: percentage and range
Sex	
Male	3 (9%)
Female	30 (91%)
Age, mean	44.32 (18–79)
BMI, mean	27.99 (16.5–48.9)
Smoking	
Smoker	0
Non-smoking	20 (60%)
No data	13 (42%)
Respiratory diseases	
Asthma	5 (15%)
COPD	0 (0%)

#### Surgical settings

All cases had been treated endoscopically with balloon dilatation and adjuvant injection of corticosteroids in the stenotic lesion. Treatment was done under general anaesthesia, and patients were assessed peri-, operatively using rigid suspended laryngoscope and ventilated with superimposed supraglottic ventilation utilizing jet ventilation (Twinstream ref: CTNS-110-000). The procedures were conducted with 2–3 small incisions in the stenotic area in the shape of a star using a sharp cold steel knife. For diagnostics a tissue sample of 2 × 2 mm was taken, followed by approximately 0.4–0.5 ml of methylprednisolone injected in the lesion. Finally, the balloon was placed and dilation for approximately 2 min in apnoea was conducted. The balloon used was a Boston scientific pulmonary balloon dilatation catheter 12–15 mm diameter 3 cm long (ref M00550350, up to 8 atm pressure) or 15–18 mm diameter 5.5 cm long (ref M00550310, up to 7 atm pressure). The operative procedure generally was conducted over 20–30 min.

#### Patient reported outcome measure (PROM)

Dyspnea index (DI) is a 10-question form specially developed for the evaluation of upper airway disorders, it has been recently translated and validated to Swedish [10, 12]. Responses gives a score 0–40 with a higher number representing more symptoms. The cutoff levels have been proposed by Gartner-Schmidt [10]. Short Form Health Survey 36 (SF 36) is questioner measuring both physical and psychological health status in a self-reported form. The questionnaire consists of 36 multiple choice questions that are categorized into 8 separate health concepts: physical functioning, role limitations due to physical health, role limitation due to emotional problems, energy/fatigue, emotional well-being, social functioning, pain, and general health. It also includes a ninth category giving an idea of the patients change in health. All categories give a value 0–100 whereas a higher number representing a better quality of life. SF 36 has been used to evaluate quality of life in patients for a very long time and is well-validated in several studies [13].

Cases were preoperatively assessed with flexible laryngoscopy the day before surgery and are asked to complete the SF 36 and DI forms. The post-operative forms were completed after 3 months often in our outpatient clinic. Due to long distances from home to hospital, some patient chose to have their follow-up by phone, and in these cases, the forms are sent to the patients by mail. The reason for using a timeframe of 3 months postoperatively is due to the common time course of SGS which is dynamic, progressive, and recurrent. Our aim is not to study SGS in a longitudinal perspective but to measure the outcome of the used surgical balloon method with robust patient reported outcome measures highlighting the patient perspective and test stability.

### Statistical analysis

All statistical analyses were performed using SPSS version 28.0.0.1. Since we have data for the same individuals pre-, and post operatively and we wish to make as few distributional assumptions as possible, significance test was performed using the Wilcoxon signed rank test.

## Results

### Study populations

The total number of research participants screened was 40 patients, as shown in Fig. 1, two patients were excluded due to inability to understand and sign the consent document, one due to blindness, one due to psychiatric comorbidity and one patient was excluded, since the age was under 18 years of age. Three patients were not interested in surgery despite being diagnosed with highly symptomatic SGS and, therefore, not included. One patient failed to fill out the post-operative forms. Finally, 33 participants entered the study and completed the pre-operatively and post-operatively assessments. None of the participants had conditions in lower airways affecting the anaesthetic procedure or the surgical procedure. There was no postoperative bleeding in any case, nor damage to tongue, teeth, or lips.

Thirty cases out of 33 (91%) were females and 3/33 were males (9%). Sixty percent (20/33) were non-smokers, in 39% (13/33) no data on smoking status was available, and no cases were active smokers. Mean BMI for the whole group was 27.99, which is defined as overweight, but not obesity, with values ranging from (16.5–48.9) (Table 1). Thirty-three participants completed the Swedish version of the DI and SF-36 forms. Five out of 33 (15%) reported asthma as defined by (i) self-report and (ii) labelled J45 diagnose (with or without supplement) according to International Statistical Classification of Diseases and Related Health Problems (ICD) and no patients were suffering from COPD.

### Histopathological findings

Out of the 33 included cases 30 was labelled with the histopathological phrase “Epithelialized soft tissue, hyalinization and chronic non-specific inflammation”. Two of these 30 cases (7%) were reported as having low-grade dysplasia. One case out of 33 (3%) was diagnosed with granulomatosis with polyangiitis (GPA) on biopsy from the SGS lesion, another SGS case was previously diagnosed and systematically treated for GPA but the histopathology from this biopsy was negative, concluding 2/33 (6%) GPA cases in our material. One case was diagnosed with leiomyoma in the SGS lesion and another with positivity for IgG4 and IgG.

### Dyspnea index and SF 36, pre vs. post operatively

**Fig. 2:**
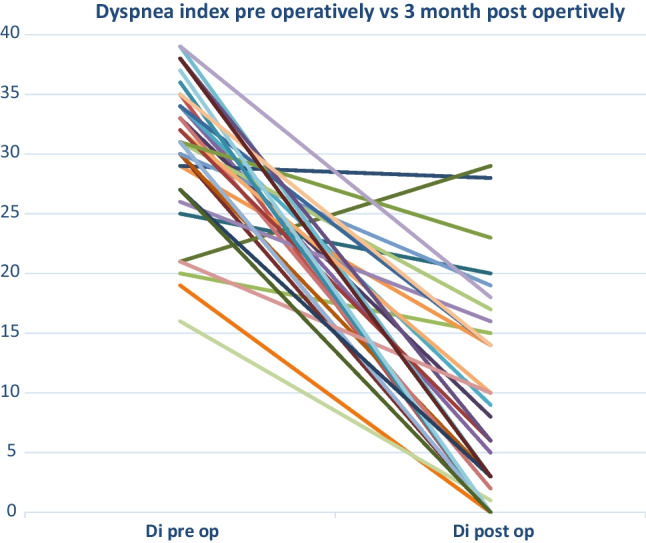
Illustrating postoperative DI (Dyspnea index) scores compared to pre-operative scores, 32 out of 33 patients improve their score

Preoperatively values for DI and SF 36 were compared with post operatively values. Median (mean also given in parentheses) DI pre-operatively was 31 (30.39), with a drop post operatively to 6 (8.97). When applying the Wilcoxon signed ranked test the pre–post difference was significant (*p* < 0.001) indicating improvement (reduced values) (when comparing pre-, and postoperative scores are illustrated in Fig. 2 and statistical results in Table 2. Only one case out of 33 (3%) reported a higher post-operative DI score compared to their pre-operative assessment.

**Table 2:** Statistical summary and significance test for DI (Dyspnea index)

	*N*	Mean	Median	25th	75th	^a^Two-tailed *p* value
Dyspnea index pre-op	33	30.39	31.00	27.00	34.50	
Dyspnea index post-op	33	8.97	6.00	0.50	15.50	**< **0.001

Table provides data on postoperative scores at group level compared to pre-operative scores using Wilcoxon signed ranked test

Scores for SF-36 pre- and post-operatively are reported separately for all 9 categories in Table 3. The value of calculating a SF-36 *total score* is a matter of debate, since it has been suggested to be a measurement bias affecting validity, we report data separately for all 9 categories [13].

**Table 3:** Statistical summaries and significance tests for SF-36 (short form health survey)

	*N*	Mean	Median	25th	75th	^a^Two-tailed *p* value
Physical functioning pre-op	33	48.03	50.00	27.50	70.00	
Physical functioning post-op	33	88.64	95.00	85.00	100.00	**< 0.001**
Role limitations due to physical health pre-op	33	31.06	0.00	0.00	75.00	
Role limitations due to physical health post-op	33	90.15	100.00	100.00	100.00	**< 0.001**
Role limitations due to emotional problems pre-op	33	59.60	66.70	0.00	100.00	
Role limitations due to emotional problems post-op	33	89.90	100.00	100.00	100.00	**0.005**
Energy/fatigue pre-op	33	36.67	35.00	20.00	50.00	
Energy/fatigue post-op	33	63.33	75.00	52.50	80.00	**< 0.001**
Emotional well-being pre-op	33	66.06	64.00	52.00	78.00	
Emotional well-being post-op	33	80.97	88.00	72.00	92.00	**0.002**
Social functioning pre-op	33	59.47	50.00	37.50	81.24	
Social functioning post-op	33	86.74	100.00	81.25	100.00	**< 0.001**
Pain Pre-op	33	74.78	90.00	45.00	100.00	
Pain Post-op	33	85.08	100.00	72.50	100.00	0.047
General health pre-op	33	56.06	55.00	40.00	72.50	
General health post-op	33	67.88	70.00	60.00	80.00	**0.003**
Health change pre-op	33	24.24	25.00	0.00	50.00	
Health change post- op	33	84.84	100.00	75.00	100.00	**< 0.001**

Scores for all nine separate categories are presented at 3 months post -operatively follow-up, compared to pre-operative scores, using Wilcoxon signed rank test. Pre-op; pre-operatively and post-op; post-operatively. *p* values in boldface survive Bonferroni correction for 9 comparisons

## Discussion

The primary findings of this study support the interpretation that endoscopic treatment of fibrotic subglottic stenosis (SGS) with balloon dilatation has led to benefit in terms of improvement of quality of life for this cohort. DI and SF 36 score improved (reduced values) significantly in all categories post-operatively, which demonstrates consistency. These findings are supported by previous reports [14, 15]. The novelty of this publication is the use of DI and SF-36 combined data in this new cohort of SGS patients in Northern Sweden.

The reason for using PROMS as measures of gains from surgery is that they are directly from the study participants for outcomes that were chosen, because they are important for patients, thus avoiding interpretation bias by caregivers/researchers. There are several studies with focus on measurements of objective functional assessment with the best discriminatory power of SGS from other respiratory conditions that mimic SGS. These methods are suggested to be effective predictors for when to offer surgical treatment [16]. These objectives need to be combined with robust patient-reported outcome measure breathing disability, DI [12] and SF-36, with assessment of physical and psychological health status in a self-reported form. In clinic SGS care, it is the patient’s experience of respiratory distress and functional impairment that guides caregivers when to treat and how to treat. In line with other studies[14, 17, 18], the detection of objective airway obstruction and patient reported disability to breath clearly reflect a discrepancy between the anatomic airway and the patients perceived status commonly observed in everyday clinical practice. These PROMS (patient reported outcomes measures) can serve as reliable measurements of treatment outcomes from a patient perspective but also measurements of airway deterioration in follow-up of subglottic stenosis, since what a positive test means is easy to understand, and there is no interpretation bias by a caregiver.

The timeframe for the post-operative outcome measures of data here was chosen at 3 months due to the natural history of SGS which is a dynamic, progressive, and recurrent condition. Evaluating different surgical techniques applied in SGS care with focus on sustainability of benefit (or harm) needs to have timeframes of perhaps several years to identify persisting long-term effects on surgical settings associated with elevated risk for recurrence [19].

### Strength and limitations

The prospective design for inclusion and assessment is a strength for limiting some forms bias which can affect results compared to purely retrospective studies. This report concerns a relatively small cohort and one center, which could be considered a weakness, since it might limit generalizability for other treatment practices, as well as recognizing that there is always risk for false positive results with small cohorts. In this case, the results were so consistent, that false positivity is unlikely [14, 15]. The consequences of this normally includes overestimates of effect size and reproducibility, however, considering previous findings with identical outcomes [14, 15], and clear-cut significance this study might confirm the reproducibility and increase the scientific value although reporting limited inclusion.

The absence of an objective disease severity grading might be considered a drawback, and as an example, the most used grading scale worldwide for this disease, the Cotton–Myer scale, does not assess SGS severity nor does it address the complexity of the lesion or cross sectional degree of SGS [3]. Moreover, Song et al. [17] confirmed poor inter-rater reliability for the visual estimation on Cotton–Mayer grading scale, and as such interpretation difficulties in contrast to the PROM’s used in this study. The lack of a routine preoperative functional evaluation with spirometry in this population might be considered another shortcoming, since there is evidence that measurements of pulmonary function that might serve as diagnostic markers in monitoring SGS [17]. We hypothesized an improvement in DI postoperatively but not in all nine categories for SF-36 and especially not in “pain categories”, since SGS is not commonly associated with pain. A minority of the patients in our study suffered from asthma and us not evaluating them as a separate group could be considered a drawback whereas one could argue that we could not be certain if our shown improvement is only a courtesy of the balloon dilatation. However, no changes were made in the patient’s asthma medications during the follow-up period so therefore we believe that the improvement in both DI and SF-36 is related to our chosen surgical treatment rather than correlated to their asthma.

## Conclusion

Endoscopic treatment of subglottic stenosis (SGS) with balloon dilatation is a beneficial method for improvement of the ability to breath and to quality of life as measured by robust patient-reported outcome measures; SF 36 (short form health survey) and DI (dyspnea index), ensuring the use of a safe and well-tolerated procedure and the benefit of the patient.

## Data Availability

The data that support the findings of this study are available from the corresponding author AE, upon reasonable request, from other investigators adhering to the European Union General Data Protection Regulation (EU) 2016/679 GDPR.
